# Significantly enhanced creep resistance of low volume fraction in-situ TiBw/Ti6Al4V composites by architectured network reinforcements

**DOI:** 10.1038/srep40823

**Published:** 2017-01-17

**Authors:** S. Wang, L. J. Huang, L. Geng, F. Scarpa, Y. Jiao, H. X. Peng

**Affiliations:** 1 School of Materials Science and Engineering, Harbin Institute of Technology, Harbin 150001, China; 2 Advanced Composites Centre for Innovation and Science (ACCIS), Bristol University, Bristol, BS8 1TR, United Kingdom; 3 Institute for Composites Science Innovation (InCSI), School of Materials Science and Engineering, Zhejiang University, Hangzhou 310027, PR China

## Abstract

We present a new class of TiBw/Ti6Al4V composites with a network reinforcement architecture that exhibits a significant creep resistance compared to monolithic Ti6Al4V alloys. Creep tests performed at temperatures between 773 K and 923 K and stress range of 100 MPa-300 MPa indicate both a significant improvement of the composites creep resistance due to the network architecture made by the TiB whiskers (TiBw), and a decrease of the steady-state creep rates by augmenting the local volume fractions of TiBw in the network region. The deformation behavior is driven by a diffusion-controlled dislocation climb process. Moreover, the activation energies of these composites are significantly higher than that of Ti6Al4V alloys, indicating a higher creep resistance. The increase of the activation energy can be attributed to the TiBw architecture that severely impedes the movements of dislocation and grain boundary sliding and provides a tailoring of the stress transfer. These micromechanical mechanisms lead to a remarkable improvement of the creep resistance of these networked TiBw/Ti6Al4V composites featuring the special networked architecture.

Titanium alloys, and in particular titanium matrix composites (TMCs) have been evaluated in board range of aerospace, weapons and sports equipment applications^1^,^2^,^3^. The main rationale behind their use is the high strength-to-weight ratio and high corrosion resistance^4^,^5^. TMCs have also been used to produce composite monofilament lattice structures^6^ and composites with carbon fiber reinforcements for nuclear applications^7^. Quite recently TMCs have also been manufactured via selective laser melting for biomedical applications^8^,^9^,^10^. Discontinuously reinforced titanium matrix composites (DRTMCs) have also been developed, and some examples of this class of metal matrix composites are TiB/Ti^11^, TiC/Ti-6Al-4V^12^, (TiB + TiC + La_2_O_3_)/Ti^13^ and (Ti_5_Si_3_ + TiB)/TC4^14^ compounds. Among the various reinforcements adopted for DRTMCs, TiB whiskers (TiBw) are considered to be one of the best because of their high strength and good chemical compatibility with the titanium matrix^15^. Recently, Huang *et al*.^16^ have developed a class of TiBw/Ti6Al4V composite with a network microstructure in which the reinforcements have a designed and tailored network-like distribution, instead of a conventional homogeneous one. These composites show superior high-temperature tensile properties over their traditional TMC counterparts.

The understanding and tailoring of the creep performance are preconditions for an effective use of TMCs in structural applications at high temperatures^11^,^17^,^18^,^19^. The creep behavior mechanisms of traditional TMCs with homogeneous reinforcement distributions have been so far extensively investigated^2^,^17^,^20^,^21^. In general a high volume fraction of the reinforcement can increase the creep resistance, but has detrimental consequences on the room-temperature plasticity and deformability of these composites. TMCs tend to exhibit an improved creep resistance compared to analogous matrix alloys when they possess higher activation energies and stress exponents and lower steady state creep rates. The higher values of stress exponents and activation energies are in general attributed to their threshold stress (σ_0_)^2^,^19^,^22^,^23^,^24^,^25^,^26^, without which the stress exponents of the TMCs would be equal to the corresponding values of the matrix alloys. Within the temperature range of 873 K–923 K Ma *et al*.^2^,^17^ have however observed the disappearance of the threshold stress in *in-situ* 15 vol.% TiB/Ti and *ex-situ* 15 vol.% TiC/Ti6Al4V composites. Another possible explanation of the enhancement of the activation energies is related to the stress transfer from the low modulus matrix to the high modulus reinforcements^19^,^25^,^26^,^27^. According to Park *et al*.^28^ the load transfer effect can be used to explain the abnormality of the activation energy between SiC-6061Al and its 6061 Al matrix. The stress exponents have been observed to change with the varying applied stress, a likely indicator of the variation of the creep mechanisms in different stress regions. Nie *et al*.^29^ have reported the increase of the stress exponent for Ti–5Al–5Mo–5V–1Fe–1Cr alloys from 4.3–4.8 at lower stress, to 8.2–8.5 under higher stresses at a temperature of 773 K. Xiao *et al*.^19^,^25^ have investigated the creep behavior of *in-situ* TiB plus La_2_O_3_ reinforced TMCs between 873 K and 973 K, and then used a power-law constitutive equation for the steady state creep of these composites. The results suggest that the reinforcements present in the TMCs have a strong influence on the two micromechanical mechanisms that govern the creep deformation: the grain boundary sliding that dominates in the low stress region, and the dislocation climbing prevailing in the high stress region. Dastidar *et al*.^30^ have evaluated the creep deformation of Ti-8Al-1Mo-1V at 728 K, and observed that although slip was present, the grain boundary sliding was the most active deformation mechanism. In the case of Ti–5Al–5Mo–5V–1Fe–1Cr alloys the creep process has been however shown to be controlled by dislocation slip at 673 K, and by dislocation climb at 773 K^31^. Surface modifications such as the Nitrogen high-temperature plasma based ion implantation and the surface plasma carburizing process have also been demonstrated to be an effective way to improve the creep resistance of Ti-6Al-4V alloys^32^,^33^. The addition of chemical elements such as silicon can also contribute to the increase of the creep resistance^34^.

The creep behaviors of TMCs appear to be related to the volume fraction of the reinforcement^18^, as well as to the grain size of the matrix alloy. In TiB/Ti composites Tsang *et al*.^20^ observed that the steady state creep rates of the TMCs decreased dramatically with increasing volume fractions of the TiB reinforcement. The bigger the grain size of the matrix alloy and width of α + β colonies are, the lower the steady state creep rates appear to be in ref. 35. There is however a quite limited amount of available open literature about the creep behavior and related micromechanical mechanisms of *in-situ* TiBw/Ti6Al4V composites with inhomogeneous microstructure. This lack of knowledge hampers the widespread use of these novel metal matrix composites in high temperature applications.

In this work we tailor the distribution of TiBw in composites to increase the creep resistance without using high volume fractions of reinforcement. The *in-situ* TiBw/Ti6Al4V composites we produced are fabricated by powder metallurgy^36^. The parameters influencing the creep behavior are the network size and the volume fraction of the reinforcement in the network boundary. The fundamental micromechanical mechanisms involved in the creep deformation are evaluated through short-term creep tests performed at temperatures ranging between 773 K and 923 K, and applied stresses from 100 MPa to 300 MPa. The results show the presence of a radical enhancement in creep resistance of these metal matrix composites with inhomogeneous microstructure when compared to their counterpart monolithic Ti6Al4V alloys. We also provide a description of the fundamental microstructure mechanism that underpins this remarkable behavior, which can be used as a design guideline to produce high-performance metal matrix composites with networked architecture.

## Results and Discussions

### Microstructure

**Figure 1:**
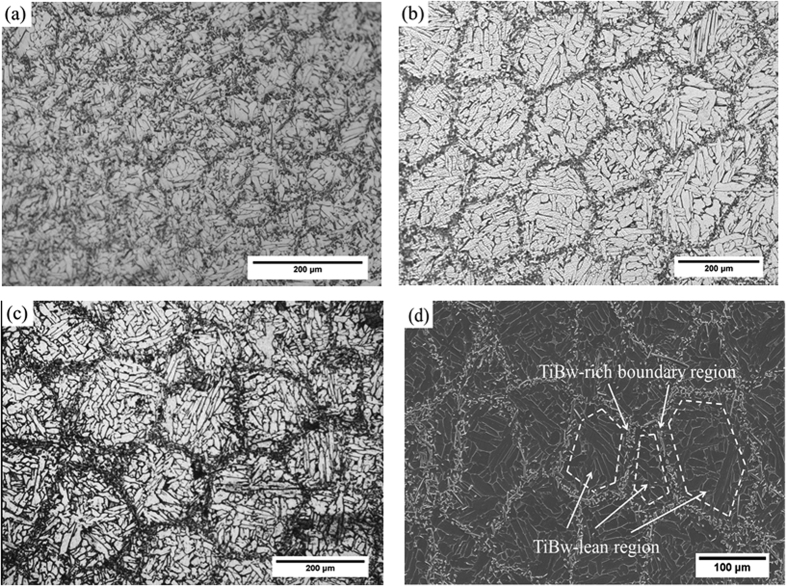
Microstructure morphology of the composites: (**a**) 8 vol.% TiBw/Ti6Al4V (65 μm); (**b**) 5 vol.% TiBw/Ti6Al4V (150 μm); (**c**) 8 vol.% TiBw/Ti6Al4V (150 μm); (**d**) Cells of a typical network microstructure at 8 vol.% TiBw/Ti6Al4V (150 μm).

The microstructure of the TiBw/Ti6Al4V composites shows the presence of a network reinforcement architecture (Fig. 1a–d). The profile of the matrix structure is near-equiaxed, and TiBw are distributed around Ti6Al4V powders forming a network microstructure similar to a cellular configuration. The formation of the network structure can be explained by considering two aspects. The first is related to the relative size of powders: compared to the Ti6Al4V particles, the TiB_2_ powders are very small, therefore they can only adhere to the surface of the large Ti6Al4V particles. The size and shape of the Ti6Al4V particles do not also change because of the low-energy ball-milling operated. The second aspect to consider is related to the metallurgical process adopted. The solid state powder metallurgy ensures that the reaction between Ti and TiB_2_ only occurred on the Ti6Al4V particle surface. The grain size of the network cell is determined by the size of the Ti6Al4V particles, and the TiB whiskers around the Ti6Al4V particles have constrained during the powder metallurgy because of the network microstructure. Therefore, the grain size of matrix particles does not change significantly. By inspection of Fig. 1a–c the size of the network cells is on average close to nearly 65 μm and 150 μm as predicted. The overall volume fractions of the reinforcement increase with the increase of local volume fraction of the TiBw within the network region (Fig. 1a–c). It is also true that the matrix is penetrating through the network boundary (Fig. 1d). One can note that in a typical network cell there are two regions: an internal one where few whiskers exist (TiBw-lean region), and the boundary that is a TiBw-rich region. Because of the different volume fractions of the TiBw and their relative locations, the creep behavior of these two regions will be different.

By observing Fig. 1a–d and other microstructure graphs, no voids are present. The lack of voids indicates that the composites are very dense after the powder metallurgy process. The density of the composites can be measured by Archimedes method. To estimate the porosity of this type of composite, it is assumed that the theoretical density of the composite is the same of the Ti6Al4V, because the density of the Ti6Al4V matrix is almost equal to the one of the TiB whiskers (4.51 and 4.52 g/cm^3^ respectively). Therefore, the porosities of the composites produced are in general less than 1%.

**Figure 2:**
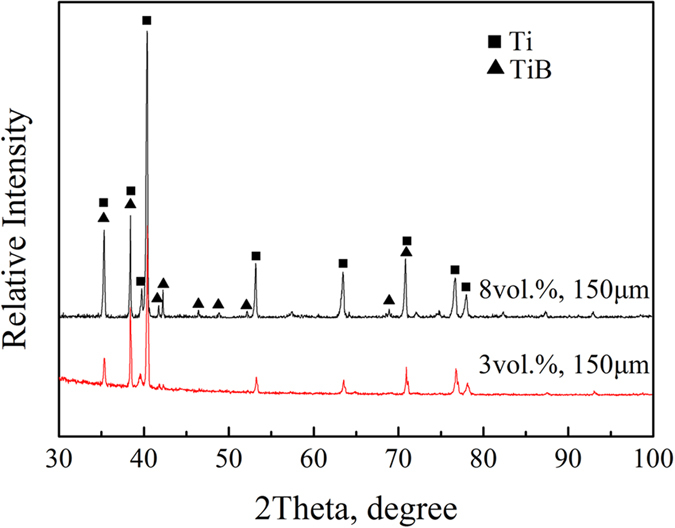
X-ray diffraction patterns of the fabricated composites.

To confirm that the reaction between Ti and TiB happens and ensure that no TiB_2_ is left we have performed X-ray diffraction. Figure 2 shows the diffraction patterns relative to the 8 vol.% TiBw/Ti6Al4V (150 μm) and the 3 vol.% TiBw/Ti6Al4V (150 μm) composites. The patterns confirm that only Ti and TiB are present in the composites. The TiB_2_ phase is not present, which indicates Ti and TiB_2_ react completely. A similar result related to analogous composites made with selective laser melting can be found in ref. 4.

### Creep behavior

**Figure 3:**
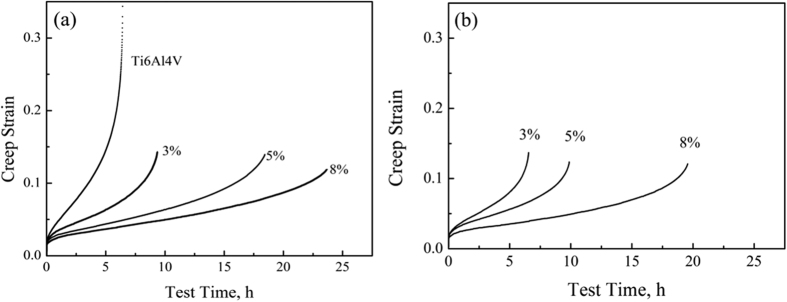
Creep behaviors of the Ti6Al4V alloy and 3 vol.%, 5 vol.% 8 vol.% TiBw/Ti6Al4V composites tested at 873 K and 200 MPa: (**a**) network size of 150 μm; (**b**) network size of 65 μm.

Figure 3 shows the creep behaviors of the monolithic Ti6Al4V alloy and the TiBw/Ti6Al4V composites with different volume fractions of reinforcement when tested at 873 K and 200 MPa. The materials share the same network sizes of 65 μm and 150 μm, respectively.

It is noticeable that the monolithic Ti6Al4V alloy and the TiBw/Ti6Al4V composites show a similar and typical creep process. One can observe the presence initially of a primary creep stage, during which the creep rate decreases with the deformation time. This phase is then followed by a steady state creep stage with steady creep rate, followed by an accelerating creep phase after which the sample fails. Among these three stages, the steady state creep one is the most important. The creep rate during this stage is generally considered for the evaluation of the creep properties and resistance of materials. The creep rates of the TiBw/Ti6Al4V composites are much smaller than the ones of the monolithic Ti6Al4V alloy during the steady state creep stage, and for 8 vol.% composites (150 μm) the creep rate is nearly of an order of magnitude lower (Table 1). This feature indicates that the creep resistance of the TiBw/Ti6Al4V composites is significantly higher than the one of the monolithic alloy. A similar enhancement of the creep resistance given by the presence of reinforcements could also be observed in traditional TMCs with homogeneous microstructure^20^. Figure 3 shows however that the improvement in creep resistance of the TiBw/Ti6Al4V composites with networked microstructure is much more evident: for a 8 vol.% composites (150 μm), the creep time before fracture reaches a 400% improvement compared to the one of the monolithic Ti6Al4V alloys. A similar result can be also found in ref. 20, while the monolithic material is Ti and the volume fraction is 15 vol.%. Even when the composites are reinforced with low volume fractions of TiBw (3 vol.%) the creep time before fracture is twice the one of the monolithic alloy. The latter aspect suggests that the volume fraction of the reinforcement is not the sole agent for the dramatic improvement in the creep performance during the steady state stage in these TiBw/Ti6Al4V composites with networked microstructure. From observing Fig. 3a and b, one can notice that the steady state creep rates decrease dramatically with increasing volume fraction of reinforcements. However, the creep strains of the composites are similar (about 0.15) for all the different volume fractions of the reinforcement and the different network sizes used. Moreover, a remarkable extension of the creep time before fracture is observed in Fig. 3a and b for the 5 vol.% TiBw/Ti6Al4V composites (twice as long as the of the 3 vol.% TiBw/Ti6Al4V samples). This phenomenon further confirms the possibility that TiBw distributed with a networked morphology have a strong effect on the creep properties. From these results, a composite with TiBw volume fractions higher than 8 vol.% may possess an even lower steady state creep rate. However, the ductility of the composite with higher volume fractions will be also lower^37^, which may jeopardize its value of engineering applications.

**Figure 4:**
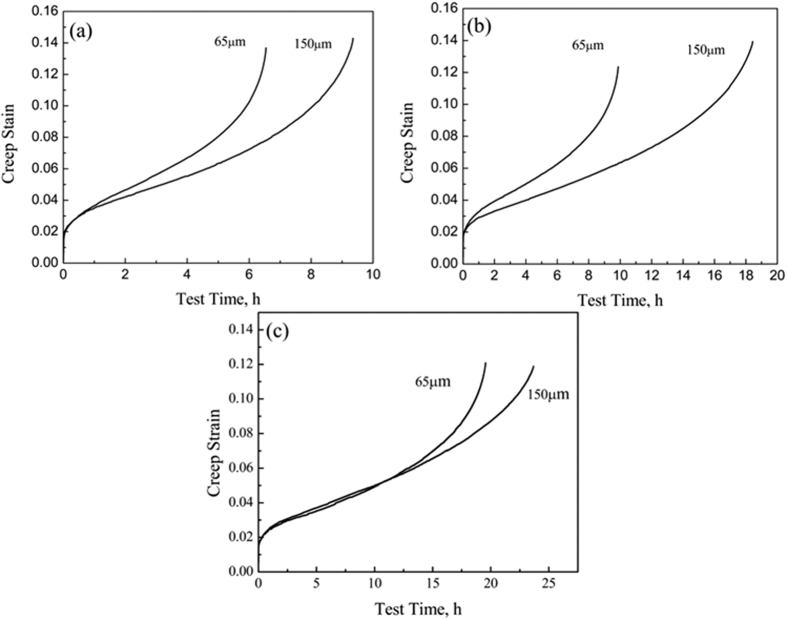
Creep behavior of (**a**) 3 vol.%; (**b**) 5 vol.% and (**c**) 8 vol.% TiBw/Ti6Al4V (65 μm and 150 μm) composites tested at 873 K and 200 MPa.

The effect of the network size on the creep behavior of the TiBw/Ti6Al4V composites with equal volume fraction is shown in Fig. 4. The steady state creep rates decrease significantly with increasing network sizes, while the creep strain still remains substantially unchanged, leading to a remarkable extension of the creep time. A similar trend is also present in the TiBw/Ti6Al4V composites, but this time the steady state creep rates decrease with increasing volume fractions of the reinforcement. This phenomenon could be attributed to the increasing local volume fractions of the reinforcement in the TiBw-rich network boundary: although the two TiBw/Ti6Al4V composites (65 μm and 150 μm) share equal overall volume fractions of TiBw, the local volume fraction of TiBw in the network boundary is higher in the composites with larger network size (Ti6Al4V powder size). The reason for this difference in local whisker volume fraction is that larger powder sizes provide smaller specific surface area. When the overall volume fractions of the TiBw are the same, the local whisker volume fraction of the composites with larger powder size must be higher than that of the composites with smaller powder size. Compared to a composite with a network cell size of 150 μm, the TiBw in composites with 65 μm tends to be uniformly distributed, which also proved that tailoring distribution is an effective way to increase the creep performance of TMCs.

To evaluate the local volume fractions of the TiBw inclusions more accurately, we have used the following equation^38^:


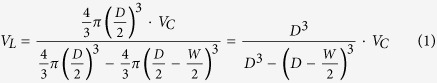


where V_L_ is the local volume fractions of TiBw, V_C_ is the overall volume fractions of TiBw, D is the average network size, and W is the average width of the TiBw-rich boundary region. To simplify the calculation, W is regarded as having the same value independently of the overall volume fractions and the network sizes. The edge of the TiBw is detected using an image data processing software (Image J). The widths of the TiBw-rich boundary regions are measured to be close to a value of 20 μm. Using equation (1), the local volume fractions for the six types of composites have been calculated in Table 2. The local volume fractions of the whiskers increase with the increase of the network size.

In conclusion, the increasing size of the networked structure leads to similar effects provided by local increases of the volume fraction of the TiBw. This is a rather different mechanism from the one present in traditional TMCs in which the reinforcements are uniformly distributed. In addition, the TiB whiskers are supposed to provide a constraint effect on the deformation of the Ti6Al4V matrix, and are responsible for delivering a suitable stress transfer during the creep process. Dislocations also tend to accumulate around the networked reinforcements and contribute to the presence of multiple cracks on the TiBw regions.

**Figure 5:**
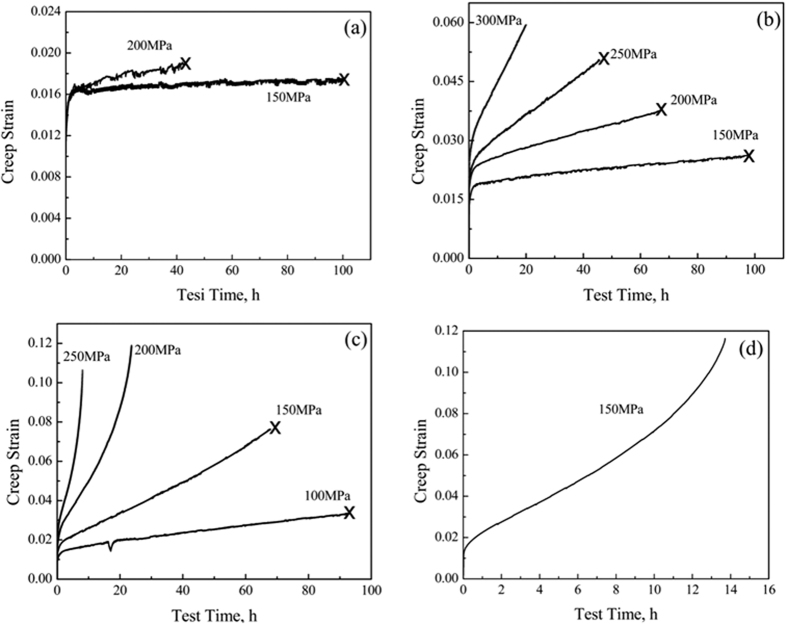
Creep behavior of the 8 vol.% TiBw/Ti6Al4V (150 μm) composites when tested at: (**a**) 773 K, (**b**) 823 K, (**c**) 873 K and (**d**) 923 K.

From the above analysis it can be concluded that the TiBw/Ti6Al4V composites with the maximum volume fraction of TiBw and the largest network size provide the highest creep resistance. We have therefore further investigated the creep behaviors of the 8 vol.% TiBw/Ti6Al4V (150 μm) composites to discern the creep mechanisms of these composites. Figure 5 shows the creep behaviors of the composites tested for different parameters (temperature range of 773 K–923 K and stresses range of 100 MPa-300 MPa). The steady-state creep stage of the composites last for quite a long time at low temperature and stress, therefore the creep tests have been terminated after obtaining the steady state creep rates (tagged in Fig. 5).

**Figure 6:**
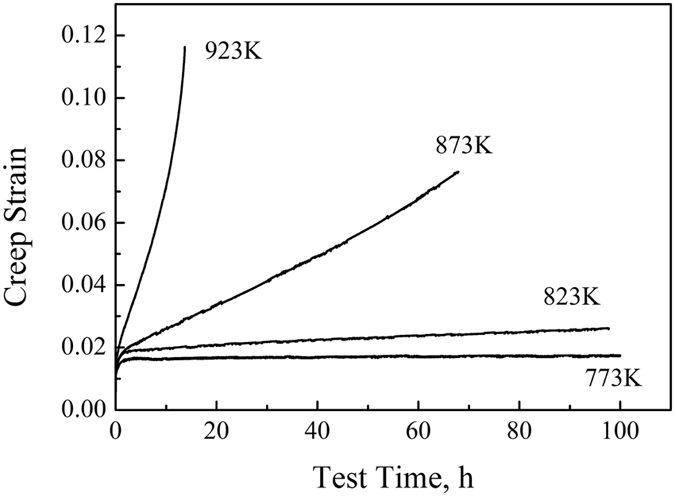
Creep behaviors of the 8 vol.% TiBw/Ti6Al4V (150 μm) composites under the same stress 150 MPa and different temperature (range of 773 K–973 K).

The influence of the temperature on the creep behavior of the composites at 150 MPa is illustrated in Fig. 6. The results clearly show that the steady state creep rate decreases dramatically with decreasing temperature, which indicates a clear increase of the creep resistance.

**Figure 7:**
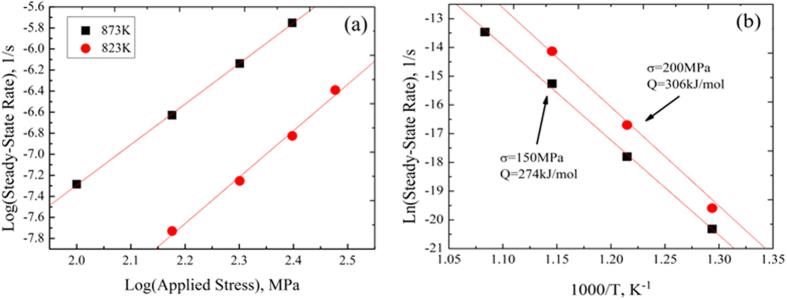
The identification of the creep parameters: (**a**). Stress exponent at 823 K and 873 K; (**b**). Activation energy at 150 MPa and 200 MPa.

The creep rate during the steady state stage is related to the applied stress and the deformation temperature, and it could be described by a power-law relationship^2^,^26^:


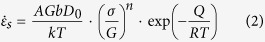


where 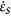 is the steady state creep rate, *A* is a constant of material, *G* is the shear modulus, *b* is the Burgers vector, *D*_*0*_ is the diffusion coefficient, *k* is the Boltzmann constant, *T* is the Kelvin temperature, *σ* is the applied stress, *n* is the stress exponent, *Q* is the activation energy and *R* is the gas constant. According to equation (2) the stress exponent could be calculated by measuring the slope of the 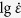-lg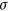 curves. From the results shown in Fig. 7a the stress exponents of the TiBw/Ti6Al4V composites are 4.4 at 823 K and 3.9 at 873 K. These values are close to the ones measured in diffusion controlled dislocation climb processes^20^. The stress exponents of the composites described in this work are lower compared to the ones in traditional TMCs (more than 5)^2^,^19^,^25^, but are in agreement with the stress exponents of monolithic Ti6Al4V (from 4.1 to 4.4)^39^. This fact can be explained by the contribution from the TiBw-lean region (inner network boundary) during the take up of the strain, which is similar to the one of the Ti6Al4V alloy. Consequently, no threshold stress can be observed in the TiBw/Ti6Al4V composites. Figure 7b shows the calculation process associated to the activation energy of the composites within the temperature range between 773 K to 923 K. The activation energy of the TiBw/Ti6Al4V composites is 274 kJ/mol when the stress is 150 MPa, and 306 kJ/mol when the stress reaches 200 MPa. These values are consistent with those present in traditional TMCs, and significantly higher than the ones in *a*-Ti materials (less than 241 kJ/mol^40^). The increase of the activation energy indicates a substantial enhancement of the creep resistance. It is apparent from the above results that both the content and the distribution of the TiB whiskers enable to enhance and tailor the creep performance of the DRTMCs.

The power-law relationship could also be described using the Arrhenius equation by re-arranging equation (2):


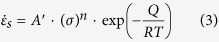



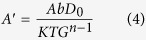


From the values of the stress exponent *n* and the activation energy *Q* extracted above, the power-law relationship for the 8 vol.% TiBw/Ti6Al4V (150 μm) composites can be expressed as follows:


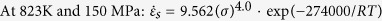



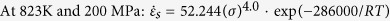



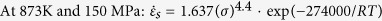



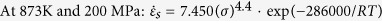


However, there are some disadvantages in applying the power-law relationship to predict the creep behaviors of materials, mainly because *Q, n* and 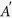 change with the variation of deformation parameters. Brnic *et al*.^41^ have proposed the following formula for creep tests:


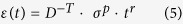


where *T* is the temperature, *σ* is the stress, *t* is the time, *D, p* and *r* are the parameters related to creep stages. These parameters are influenced more by the testing temperature than by the applied stresses^41^,^42^,^43^. To calculate the *D, p* and *r* parameters in equation (5), it is useful to re-arrange the expression as follows:


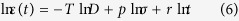


**Figure 8:**
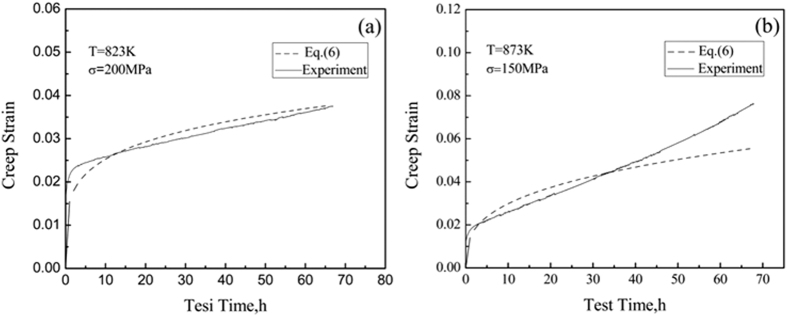
Comparison between simulated and experimental creep curves for the TiBw/Ti6Al4V composites at (**a**) 823 K, 200 MPa and (**b**) 873 K, 150 MPa.

The values of the identified parameters *D, p* and *r* are listed in Table 3. These values are used to reconstruct the theoretical creep curves according to equation (6), which are shown in Fig. 8 along with the corresponding experiment results. The discrepancy between the experimental results and the simulation decreases with longer testing times during the steady state creep stage (from 10 h to 40 h in our case). Figure 8b also shows that the experimental curve generally deviates from the theoretical one when the test time is longer than 50 h, which could be mainly attributed to altering creep stages, from the steady state to the accelerating one. It is however apparent that the Brnic *et al*. model can be applied to predict in an adequate manner the creep behaviors of the TiBw/Ti6Al4V composites with the networked microstructure.

### The creep mechanism of the network architecture and the local TiBw

**Figure 9:**
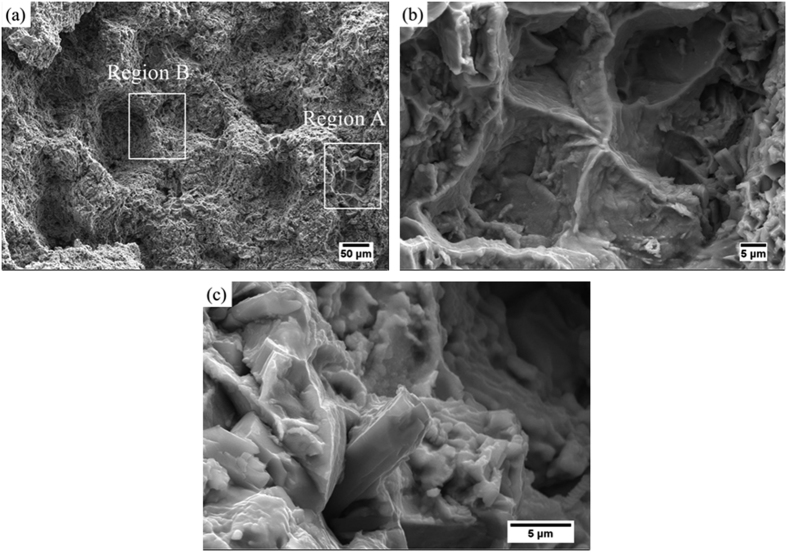
Fracture morphology of the 8 vol.% TiBw/Ti6Al4V (150 μm) composites after the creep test carried out at 873 K and 200 MPa. (**a**) Fracture surface; (**b**) magnified morphology of the region A; (**c**) magnified morphology of the region B.

The topology of the microstructure provides a particularly strong effect on the creep behavior of materials^44^,^45^,^46^. Figure 9 shows the fracture morphology of the 8 vol.% TiBw/Ti6Al4V (150 μm) composites after creep tests performed at 873 K and 200 MPa. Although the typical fracture morphology of the Ti6Al4V matrix^47^ in region A can be well noticed (Fig. 9b), the majority of the fracture took place at the network boundary with high local volume fraction of TiBw. During the creep process the dislocation slip first occurs in the TiBw-lean region, with subsequent generation of macro-strain. The dislocation slip is severely hampered when it tends to move to the TiBw-rich network boundary where the local volume fraction of TiBw is high, and this give rise to a high creep resistance. On a lower scale, the fracture morphology suggests that the creep failure might be attributed to debonding between the TiB whiskers and the Ti6Al4V matrix. Figure 9c shows the magnified morphology of fracture surface in the B region. The TiBw extracted from the matrix alloy indicates that during the primary creep process the whiskers prevent the micro-cracks from spreading. In addition, the whiskers around the Ti6Al4V matrix provide a constraining effect on the matrix itself during the creep process, leading to a low deformation of the Ti6Al4V alloy before the debonding of the TiBw. The constraining effect appears to be linked with the increasing dislocation density. The dislocation tends to accumulate around the TiBw, leading to the debonding between the TiBw and the Ti6Al4V and the failure of the reinforcements. Overall, the presence of the TiB whiskers leads to an improvement in creep resistance of the composites, accompanied by a decreasing creep strain. The TiBw act like a shell around the Ti6Al4V matrix: when the reinforcements cannot sustain extreme values of stress the whiskers fail first and act as a sacrificial buffer.

**Figure 10:**
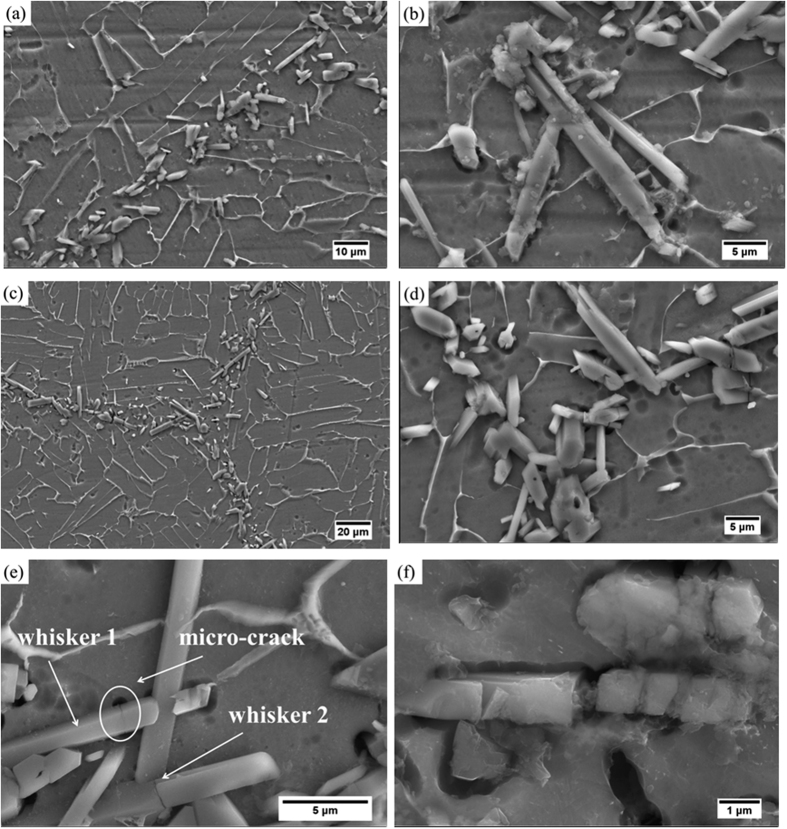
Evolution of the microstructure of the 8 vol.% TiBw/Ti6Al4V (150 μm) composites during creep tests carried out at 873 K and 200 MPa. (**a**) and (**b**) after 2 hours; (**c**) and (**d**) after 10 hours; (**e**) and (**f**) after failure.

The evolution of the microstructure of the TiBw/Ti6Al4V composites during creep is further investigated to reveal other potential aspects of the function of the TiBw on the creep behaviors of these networked composites. Figure 10a and b show the morphology of a specimen crept for 2 h (creep strain 0.029). The initial microstructure of the composites (see Fig. 1) remains unchanged and no cracks or voids are formed during this period. This phenomenon indicates the role of the networked microstructure to stabilize the deformation, and proves that the TiBw-lean region can share strain - i.e., it creates dislocation slip. When the creep test is performed for 10 h (creep strain 0.043), the morphology of the specimen is displayed in Fig. 10c and d. In this case one can observe the presence of multiple micro-cracks on the TiBw network boundary. It is also possible to note the debonding effect existing between the TiBw and Ti6Al4V alloy. Cracks and debonding at this stage are still however limited because of the constraining effect of the TiBw on debonding. As a result, the dislocation cumulates around the TiBw and leads to a high dislocation density. Figure 10e and f show the morphology of the specimen after failure. The dislocation density increases significantly because the considerable numbers of TiB whiskers are failed. A micro-crack could be observed on whisker #1 of Fig. 10e, however the crack does not extend because of the surrounding Ti6Al4V matrix blunt micro-crack that blocks the process. Although the volume fractions of whiskers on the network boundary are high, it is apparent that the TiBw reinforcement forms a quasi-continuous but not compact shell, which implies that each TiB whisker is surrounded by a ductile Ti6Al4V matrix. When micro-cracks form in the TiBw and tend to extend to the Ti6Al4V matrix plastic deformation occurs, and the stress concentration at the crack tip is released to blunt the micro-crack. The ductile Ti6Al4V matrix is instrumental to block the TiBw from extending, finally resulting in the debonding between the whiskers and the matrix. Another whisker (#2) can also be observed in Fig. 10e. Although the whisker is split in two, the fragments do not separate completely, and this can be attributed to the high value of the local volume fraction of whiskers in the TiBw-rich network boundary. From Fig. 10e it is also apparent that the whiskers are connected with each other. When whisker #2 tends to separate into two segments other whiskers impede the deformation of this inclusion and tend to slow down the crack growth. This fact explains the dramatic steady state creep rate decreases with the increase of the local volume fraction of the whiskers.

It is worthwhile pointing out that the TiBw can also fracture into several parts, as showed in Fig. 10f. This phenomenon indicates that the TiBw can efficiently undertake and transfer stress even after cracking into small fragments, which is something also beneficial to increase plasticity. During the creep process when micro-cracks appear on the TiBw region and the stress concentration exceed the tensile strength of the TiBw the whisker would crack for the first time. Due to the strong interface generated by the *in-situ* synthesis process and the networked microstructure the Ti6Al4V matrix and the adjacent whiskers can still transfer stress to the failed whisker. Therefore, the failed whisker inclusion can still undertake stress and fracture again in other places. In consequence, the networked microstructure of the composites allows a more effective stress transfer from the Ti6Al4V matrix and TiBw to other whiskers, compared to traditional TMCs with homogeneous microstructure. This feature makes the TiBw/Ti6Al4V composites to be less sensitive to the applied stress than traditional TMCs, and provides smaller stress exponents (Fig. 7a) and good ductility.

**Figure 11:**
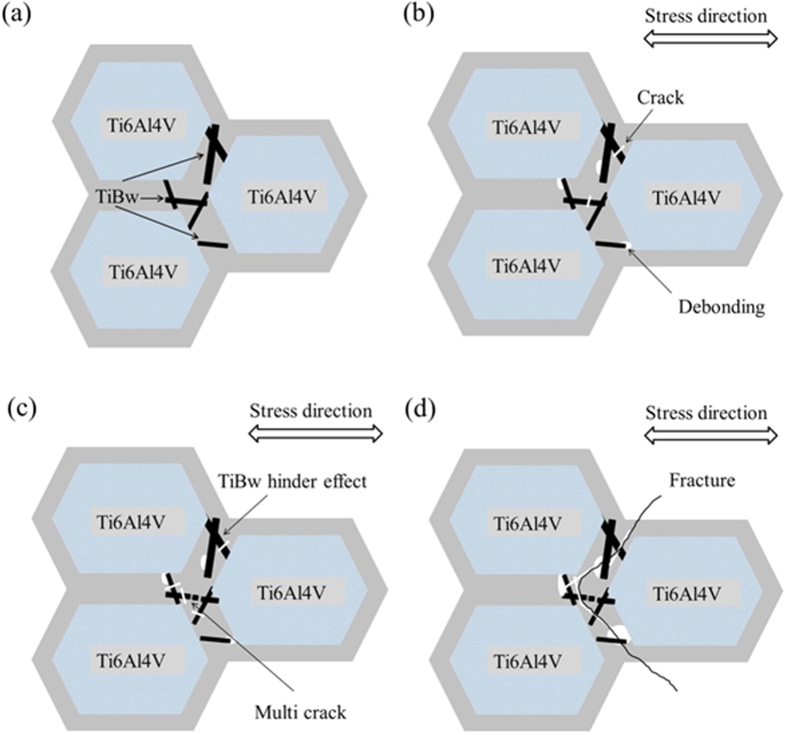
Schematic illustration (from a to d) that shows the fracture mechanism of the TiBw/Ti6Al4V composites with networked microstructure during the creep process.

A scheme showing the process of creep failure for the TiBw/Ti6Al4V composites with the networked microstructure is presented in Fig. 11. When the stress is applied to the material both the TiBw rich and lean regions resist the load, and deformation happens on the latter regions first. As the process continues crack and debonding appear on the whiskers. Because of the constraining effect provided by the other TiBw the cracks grow slowly, and multi-fracture of the TiBw start to happens. Finally, fracture occurs with the expansion of the debonded regions and the growth of the cracks on the boundary of the network with high local volume fractions of TiB whiskers.

### Materials and Methods

In this study we have used Ti6Al4V spherical particles with two different sizes: one ranging between 40~90 μm (average size of 65 μm), the other varying from 120 to 180 μm (average size 150 μm). The TiB whiskers are *in-situ* synthesized using the reaction between Ti and TiB_2_ powders with an average size of 3 μm. The adhesion of the TiB_2_ powders to the Ti6Al4V particles is provided by low energy ball milling, operated through a planetary ball mill and speed of 200 r/min for 5 hours. The mixed powders have then been hot pressed in vacuum at 1473 K for 1 h, during which period the TiB_2_ powders have reacted with the Ti according to the following reaction:


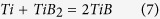


Previous work^48^ has demonstrated that TiBw can be obtained from combining Ti and TiB_2_ through powder metallurgy. The TiBw/Ti6Al4V composites with the two different network sizes (65 μm and 150 μm) and three different overall volume fractions of TiBw reinforcement (3 vol.%, 5 vol.% and 8 vol.%) have been made. The overall volume fraction of the TiBw whiskers in the TiBw/Ti6Al4V composites can be determined via the weight percentage of TiB_2_ and Ti6Al4V raw materials using the following equation^37^.


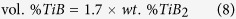


From equation (8), the 3 vol.%, 5 vol.% and 8 vol.% composites are fabricated by adding 1.77 wt.%, 2.94 wt.% and 4.71 wt.% TiB_2_ powders respectively. The local volume fraction of the TiBw reinforcement can be however determined by the network size and the overall volume fraction. For a comparative study, monolithic Ti6Al4V alloy samples have been prepared following the same process. The microstructure morphology of the composites is characterized by using an optical microscopy (OM, Olympus PMG-3) and a scanning electron microscopy (SEM, Quanta 200FEG). The specimens are characterized by X-ray diffraction (XRD, Philips X’Pert). The samples are prepared via grinding, polishing and etching in the Kroll’s reagent (5 vol.% HF + 15 vol.% HNO_3_ + 80 vol.% H_2_O). The gauge dimensions of the tensile creep specimens are 2 mm × 5 mm × 10 mm. The creep experiments have been performed using the RWS50 constant-load creep test machine, at temperatures of 773 K, 823 K, 873 K and 923 K with stresses corresponding to 100 MPa, 150 MPa, 200 MPa, 250 MPa and 300 MPa. The testing temperature is controlled within ±2 K by using three separate thermocouples placed at the middle and two ends of the test specimens.

### Conclusions

1. The steady state creep rates of the TiBw/Ti6Al4V composites with network microstructure are lower than that of monolithic Ti6Al4V and the creep rates decrease with increasing local volume fractions of the TiBw reinforcement.
2. The stress exponents (3.9 and 4.4) of the composites are in line with those of monolithic Ti6Al4V because the TiBw-lean region (inner network boundary) plays a key role in receiving the strain. The stress exponents suggest that the diffusion controlled dislocation climb process is the dominating creep mechanism of these network structure composites. The activation energies are significantly higher than the ones present in Ti alloys, an indicator of higher creep resistance.
3. The TiBw reinforcement with the network distribution constrains the deformation of the Ti6Al4V alloy, which results in a lower creep rate. Creep fracture happens because of the cracks on the TiBw and the debonding occuring between the TiBw and the Ti6Al4V matrix in the TiBw-rich network boundary.
4. Micro-cracks tend to not propagate because the high plasticity of Ti6Al4V alloy around the whiskers prohibits the TiBw from extending. Parts of the cracked TiBw do not separate because other whiskers of the networked microstructure impede the movement of the failed whisker, therefore slowing down the crack growth. During the creep process the whiskers tend to suffer multiple cracks, which indicates the applied stress can be transferred in an effective manner.

## Additional Information

**How to cite this article**: Wang, S. *et al*. Significantly enhanced creep resistance of low volume fraction in-situ TiBw/Ti6Al4V composites by architectured network reinforcements. *Sci. Rep.* **7**, 40823; doi: 10.1038/srep40823 (2017).

**Publisher's note:** Springer Nature remains neutral with regard to jurisdictional claims in published maps and institutional affiliations.

## Figures and Tables

**Table 1:** Steady state creep rates of Ti6Al4V alloy and 3 vol.%, 5 vol.% 8 vol.% TiBw/Ti6Al4V composites tested at 873 K and 200 MPa.

Network size	Overall volume fraction	Steady state creep rate
150 μm	0 vol.%	6.08 × 10^−6^ s^−1^
	3 vol.%	1.85 × 10^−6^ s^−1^
	5 vol.%	1.09 × 10^−6^ s^−1^
	8 vol.%	7.28 × 10^−7^ s^−1^

**Table 2:** Local volume fractions of the fabricated composites with different parameters.

Network size	Overall volume fraction	Local volume fraction
150 μm	3 vol.%	8.60%
	5 vol.%	14.33%
	8 vol.%	22.92%
65 μm	3 vol.%	4.49%
	5 vol.%	7.48%
	8 vol.%	11.97%

**Table 3:** The calculation results of the parameters D, p and r in equation (6).

T (K)	Applied stress (MPa)	D	p	r
823	150, 250	1.01155	0.40529	0.21243
873	100, 200	1.02119	1.66107	0.32504
